# A New Approach to the Study of Plastidial Stress Granules: The Integrated Use of *Arabidopsis thaliana* and *Chlamydomonas reinhardtii* as Model Organisms

**DOI:** 10.3390/plants11111467

**Published:** 2022-05-30

**Authors:** Fareena Rafique, Kyle J. Lauersen, Monika Chodasiewicz, Nicolás E. Figueroa

**Affiliations:** 1 Plant Science Program, Center for Desert Agriculture, Biological and Environmental Science and Engineering Division, King Abdullah University of Science and Technology (KAUST), Thuwal 23955-6900, Saudi Arabia; fareena.rafique@kaust.edu.sa (F.R.); monika.chodasiewicz@kaust.edu.sa (M.C.); 2 Bioengineering Program, Biological and Environmental Science and Engineering Division, King Abdullah University of Science and Technology (KAUST), Thuwal 23955-6900, Saudi Arabia; kyle.lauersen@kaust.edu.sa

**Keywords:** plant stress granules, chloroplast, plant stress response, *Chlamydomonas*, *Arabidopsis*, abiotic stress, liquid–liquid phase separation

## Abstract

The field of stress granules (SGs) has recently emerged in the study of the plant stress response, yet these structures, their dynamics and importance remain poorly characterized. There is currently a gap in our understanding of the physiological function of SGs during stress. Since there are only a few studies addressing SGs *in planta*, which are primarily focused on cytoplasmic SGs. The recent observation of SG-like foci in the chloroplast (cpSGs) of *Arabidopsis thaliana* opened even more questions regarding the role of these subcellular features. In this opinion article, we review the current knowledge of cpSGs and propose a workflow for the joint use of the long-established model organisms *Chlamydomonas reinhardtii* and *A. thaliana* to accelerate the evaluation of individual plant cpSGs components and their impact on stress responses. Finally, we present a short outlook and what we believe are the significant gaps that need to be addressed in the following years.

## 1. Introduction

Chloroplasts are distinctive organelles present in plants and algae. Although photosynthesis is the most prominent biological process, plastids also host several metabolic pathways that are crucial for plant physiology. Those pathways are involved in the biosynthesis of various metabolites, such as amino acids, fatty acids, phytohormones, vitamins and secondary metabolites, and in sulfur as well as nitrogen assimilation [1,2]. Chloroplasts are particularly sensitive to adverse environmental conditions, partly due to the instability of oxygenic photosynthesis and the considerable production of reactive oxygen species (ROS), even under optimal growth conditions [3,4]. Chloroplasts and their photosynthetic machinery have even been catalogued as environmental sensors that influence the expression of nuclear genes via retrograde signaling under a range of abiotic stress conditions [2,4,5,6].

Among these challenging environmental conditions, temperature increases due to climate change represent a severe threat to crop yields worldwide [7]. This is a significant limiting factor for crop production, particularly in tropical and subtropical climates [6]. Thus, the employment of modern approaches, such as molecular breeding and genetic engineering, is necessary to accelerate the development of tolerant cultivars. However, identifying entry points for molecular tools requires a comprehensive understanding of how plants respond to and recover from heat stress [6,8].

Recently, the novel field of liquid–liquid phase separation (LLPS) has gained prominence in the discipline of plant science. It is believed that proteins that have the ability to change state and aggregate in response to changes in the environment are key factors in stress signaling and signal transduction. Among these, LLPS condensates are cytoplasmic membrane-less condensates called stress granules (SGs). Stress granules are formed under stress conditions and sequester mRNA, proteins and metabolites during adverse conditions for release after favorable conditions are restored. Mutant plants with defective SG assembly or disassembly display abnormal responses under adverse conditions, which suggests that SGs play an essential role in environmental tolerance, a role that remains to be fully elucidated [9].

Notably, the recent observation and characterization of SG-like foci that form in chloroplasts of Arabidopsis (cpSGs) under heat stress conditions [10] represent a new direction in the study of chloroplast stress responses. To date, the relatively good characterization of components of the retrograde signaling pathway [11] and multiple 'omic studies [4,6,7,12] have made major contributions to piecing together the puzzle of stress responses in chloroplasts. The new field of LLPS in chloroplasts has the potential to deepen our understanding of cpSG assembly during stress and disassembly upon recovery, expanding our understanding of the stress responses in chloroplasts, and its impact on global plant stress responses.

Herein, we briefly review the current knowledge on cpSGs and critically discuss their putative physiological role in abiotic stress. In addition, we highlight the potential value of combining studies of cpSGs using *A*. *thaliana* and *Chlamydomonas reinhardtii* models as a novel approach to LLPS in plastids.

## 2. Plastidial Stress Granules

Since they were first observed and characterized [13], plant SGs have been traditionally considered membrane-less condensates that assemble in the cytoplasm through LLPS interactions. SGs are mainly composed of mRNA-ribonucleoprotein (mRNP) complexes formed from polysome disassembly as a consequence of translational repression in response to stress [9,14]. However, a recent report was the first to observe the assembly of SG-like foci inside Arabidopsis chloroplasts in response to heat stress. SG-like structures are described as foci due to their punctate forms in fluorescence imaging. This finding highlighted an unexplored field in the chloroplast biology of higher plants [10,15]. The observation of SG-like foci was followed by the development of a protocol for isolating SGs from Arabidopsis lysate and their analysis by mass spectrometry proteomics, metabolomics, and transcriptomics [10,15]. Briefly, this method combines differential centrifugation steps with affinity purification originally developed for cytoplasmic SGs [15] and which is adapted for the isolation of cpSGs [10]. It is worth mentioning that this method is very likely susceptible to the fusion of cpSGs and cytoplasmic SGs during the extraction process. Therefore, appropriate controls and data curation are critical for an accurate assignment of the proteins effectively sequestered into cpSGs.

A detailed analysis of cpSG isolates revealed that cytoplasmic and plastidial SGs share key features related to their dynamics. Both cytosolic and plastidial SG-foci can assemble quickly within a few minutes of exposure to stress and disassemble in a time-dependent manner when the stress ends. The formation of both is inhibited after treatment with a translation inhibitor that causes mRNA to stall at the polysome level. This treatment appears to block mRNA and polypeptide translocation into granules. Both SGs and cpSGs appear to have a similar structure, containing a stable core surrounded by a less concentrated but more dynamic external shell [9,10].

A proteomic analysis of cpSGs revealed the presence of 88 proteins, including two—CP29A and RIP1 (MORF 8)—that contain a prion-like domain (PrLD) and several (including CP29A) with RNA recognition motifs [10]. These features are crucial for the assembling properties of protein–RNA scaffolds [9,16]. Moreover, cpSGs were also found to contain ATPases, chaperones and, interestingly, all three subunits of the magnesium chelatase complex and some magnesium chelatase-associated proteins. Remarkably, RuBisCO activase and RuBisCO accumulation factors were also found in the cpSG isolates. In addition, RNA-seq analysis revealed the presence of plastidial transcripts encoding ribosomal proteins and ATP synthase complex subunits in cpSG-like isolated foci [10].

The fact that cpSGs sequester proteins that are essential for the photosynthetic process might be a clue to the biological relevance of cpSGs, not only for the chloroplast stress response but also for the whole plant during stress periods. The presence of similar groups of proteins have been observed in both cpSGs and cytoplasmic SGs from Arabidopsis but also in SGs from yeast and mammalian cells. These proteins include chaperones, ATPases and proteins displaying PrLD or RNA-binding motifs [9,15,17], suggesting evolutionary conservation of proteins necessary for SG assembly, dynamics and function.

## 3. cpSGs and *Chlamydomonas reinhardtii*

Although the observation of cpSGs in Arabidopsis was the first report of SGs in the chloroplasts of higher plants, the assembly of SG-like foci has also been reported in the chloroplasts of the green alga *C. reinhardtii* under high-intensity light stress [18].

Among their most exciting findings, Uniacke and Zerges [18], after testing different stresses, defined the conditions required to induce the assembly of cpSGs in the chloroplast of *C. reinhardtii* using confocal microscopy combined with chemical approaches. High light stress can produce ROS that induces oxidative stress and damage to PSII [18,19,20]. Treatment with hydrogen peroxide induced cpSGs assembly in *C. reinhardtii*, while the inhibition of PSII by 3-(3,4-dichlorophenyl)-1,1-dimethylurea (DCMU) treatment had no effect. Therefore, it can be inferred that mRNA localization to cpSGs induced by high light stress is a consequence of oxidative stress and not of damage to PSII. In addition, energy deprivation induced by chemical inhibition of ATP synthesis or by a lack of photosynthesis after incubation in the dark, phosphate deprivation, and exposure to UV light also induced the assembly of cpSGs [18]. In contrast to Arabidopsis and most model organisms in which SGs have been reported, heat shock had no effect on cpSGs formation in alga. Since it has been reported that heat stress also results in oxidative stress in *C. reinhardtii* as a consequence of ROS production [6], this observation is enigmatic. It may be caused by uncovered evolutionary aspects, unappropriated stress conditions or simply by the lack of a reliable marker for confocal microscopy. This remained the only report on cpSG-like foci until the recent report on cpSGs in Arabidopsis [10].

## 4. Combined Use of *A. thaliana* and *C. reinhardtii* as Complementary Models for the Study of cpSGs and Chloroplast Stress Response

Historically, *C. reinhardtii* has been a useful model for studying photosynthesis and chloroplast biology [21]. For most of the genes directly involved in the chlorophyll biosynthesis in vascular plants, homologs in *C*. *reinhardtii* have been identified [22]. Among all the proteins reported to localize into Arabidopsis cpSGs, chlorophyll biosynthesis-related proteins were particularly well represented; cpSGs contained all three subunits of magnesium chelatase complex CHLI1, ALB1/CHLD, and CHLH1/GUN5 and the related proteins CHLI2 and PORB [10]. All of these genes have identified orthologues in *C. reinhardtii* [22]. Therefore, it is highly probable that proteins that form cpSGs in Arabidopsis might also form cpSGs in *C*. *reinhardtii* and contribute to stress signaling/tolerance.

From another perspective, there are several examples of biological processes that were first found in *C. reinhardtii* and later studied in *A. thaliana* [21]. The observation of cpSGs is just one more case in a long and successful history of complimentary use as model organisms [21]. While both *C. reinhardtii* and *A. thaliana* have proven their value as individual models, together, they cover the evolutionary breadth of green plants, since they represent the two major subclades: Chlorophyta and Streptophyta, respectively [21,23]. Thus, studying both can reveal the similarities as well as the particularities.

*C. reinhardtii* has the following remarkable advantages over land plants in terms of studying stress responses: (i) growth conditions in liquid culture can be easily and precisely defined, (ii) heat shock stress can be separated from drought stress, (iii) stress treatment can be applied homogeneously to all cells in a culture, (iv) cultures of relatively homogeneous cells can be obtained from synchronization, and (v) gene families are smaller in Chlamydomonas compared to land plants [24]. Considering that both models diverged from a common ancestor ~1.1 billion years ago, studying the stress response in both organisms represents an opportunity to address evolutionary aspects of plant stress response [21,25].

**Figure 1:**
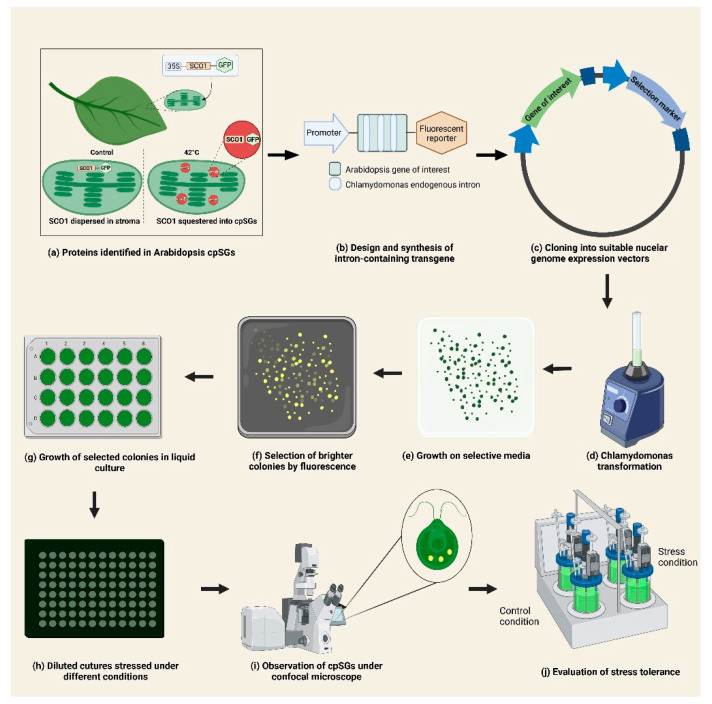
Proposed strategy for the integrated use of *A. thaliana* and *C. reinhardtii* in the study of plastid stress granules. (**a**) Selection of proteins localized into cpSGs in Arabidopsis identified by snowy cotyledon 1 (SCO1) as bait, [10]. (**b**) Selection of few candidate genes. Synthesis of their coding sequences considering codon optimization and the insertion of introns for proper expression in Chlamydomonas. (**c**) Cloning of synthesized sequences into suitable nuclear genome expression vectors (pOptimized or MoClo) [28,29]. (**d**) Transformation of *C. reinhardtii* and (**e**) growth on selective media. (**f**) Examination of positive colonies and selection based on the fluorescence of the tagged protein [31]. (**g**) Growth of selected colonies on liquid media and (**h**) dilution before stress treatment. (**i**) Evaluation of cpSGs formation by confocal microscopy [32] and (**j**) assessment of stress tolerance of transformed Chlamydomonas strains. Figure was prepared using BioRender.com.

The use of Chlamydomonas as a biotechnological chassis has not been without obstacles. It is relatively straightforward to integrate foreign DNA into the chromosomes of this alga; however, the integration proceeds by non-homologous end joining (NHEJ) in a random fashion, causing position effects on transgene expression. The reliable expression of heterologous transgenes has remained characteristically poor in this host, but has recently been overcome through advanced transgene designs that match the host gene expression regulation machinery through strategic promoter use, codon optimization, the spreading of introns, complete gene synthesis [26,27,28] and cloning into a suitable optimized nuclear genome expression vectors such as pOptimized or MoClo [29,30]. These advances in transgene design mediated by the capacity to ‘print’ customized DNA sequences have largely resolved many of the issues of reporter and target transgene expression from the nuclear genome of this host. Reporters are able to be visualized on the agar plate level by fluorescence cameras and transformants can be screened using high-throughput robotics handling techniques [31]. Fluorescently tagged proteins can be used in confocal microscopy to characterize subcellular structures in high detail [32]. *C. reinhardtii* shows significant potential as a model for the initial screening and functional characterization of *A*. *thaliana* genes involved in the assembly and dynamics of cpSGs during stress response (Figure 1).

## 5. Perspectives

The study of stress response in chloroplasts has focused on the retrograde signaling cascade and the production of ROS and physiological adaptations to counter damage to specific components of the photosynthetic apparatus. The two existing reports on cpSGs constitute good starting points for a nascent and promising field within the study of stress responses in plastids. Many key aspects, such as the varying stress conditions that trigger the assembly of cpSGs, remain to be explored. Particularly in the case of land plants, only heat stress has been reported to cause cpSG formation. Therefore, other relevant environmental stress factors faced by crops currently, such as drought and salinity, need to be investigated and the role of cpSGs elucidated. For this, employing different microscopy techniques adapted to cover the particularities of Arabidopsis or Chlamydomonas will aid in elucidating the dynamics of the assembly and the disassembly cpSGs in both models. Continued exploration of '-omics data from isolation of cpSGs will improve our understanding of the biological significance of the sequestration of those components and their specific roles in chloroplast stress response.

The combined use of *C. reinhardtii* and *A. thaliana* will play a prominent role in studying stress granules and chloroplast stress response in the following years, helping accelerate the impact assessment of individual cpSG components and unveiling unexplored characteristics of this evolutionarily conserved response. We expect that in the long term, progress on this topic contributes to identifying new targets or approaches for the development of stress-tolerant crops.

## Data Availability

Not applicable.
